# Understanding the versatile roles and applications of EpCAM in cancers: from bench to bedside

**DOI:** 10.1186/s40164-022-00352-4

**Published:** 2022-11-11

**Authors:** Yiyang Liu, Yufei Wang, Sheng Sun, Zeyu Chen, Shuai Xiang, Zeyang Ding, Zhao Huang, Bixiang Zhang

**Affiliations:** 1 Hepatic Surgery Center, Tongji Hospital, Tongji Medical College, Huazhong University of Science and Technology, Wuhan, China; 2 Clinical Medical Research Center of Hepatic Surgery at Hubei Province, Wuhan, China; 3 Hubei Key Laboratory of Hepato-Pancreatic-Biliary Diseases, Tongji Hospital, Tongji Medical College, Huazhong University of Science and Technology, Wuhan, China; 4 Key Laboratory of Organ Transplantation, Ministry of Education, National Health Commission, Chinese Academy of Medical Sciences, Wuhan, China

**Keywords:** EpCAM, Cancer, Cell signaling, Extracellular vesicles, Immune, Therapy

## Abstract

Epithelial cell adhesion molecule (EpCAM) functions not only in physiological processes but also participates in the development and progression of cancer. In recent decades, extensive efforts have been made to decipher the role of EpCAM in cancers. Great advances have been achieved in elucidating its structure, molecular functions, pathophysiological mechanisms, and clinical applications. Beyond its well-recognized role as a biomarker of cancer stem cells (CSCs) or circulating tumor cells (CTCs), EpCAM exhibits novel and promising value in targeted therapy. At the same time, the roles of EpCAM in cancer progression are found to be highly context-dependent and even contradictory in some cases. The versatile functional modules of EpCAM and its communication with other signaling pathways complicate the study of this molecule. In this review, we start from the structure of EpCAM and focus on communication with other signaling pathways. The impacts on the biology of cancers and the up-to-date clinical applications of EpCAM are also introduced and summarized, aiming to shed light on the translational prospects of EpCAM.

## Background

EpCAM is a homophilic type I transmembrane glycoprotein belonging to the small GA733 protein family [1]. Previous studies demonstrated that EpCAM participated in cell adhesion [2, 3]. However, other reports revealed EpCAM as a negative regulator of classic cadherin-mediated adhesion [4]. These inconsistent results implied the complexity of EpCAM in cancers. Apart from mediating cell adhesion, EpCAM extracellular domain (EpEX) and intracellular domain (EpICD), which can be released from membrane-bound EpCAM following TNF-α-converting enzyme (TACE)- and presenilin-2 (PS-2)-mediated cleavage, act as ligand for signal transduction receptor [5, 6] or as transcriptional cofactor [7–9]. These molecular mechanisms further border the functions of EpCAM and complicate its role in cancer progression.

Achievements in EpCAM research suggest its participation in cancer stemness, cell proliferation, metabolism, angiogenesis, epithelial-to-mesenchymal transition (EMT), metastasis, chemo/radio-resistance and immunomodulation [6, 10–15]. During tumor progression, EpCAM undergoes crosstalk with many pivotal signaling pathways, such as Wnt/β-catenin, TGF-β/SMAD, EpEX/EGFR, PI3K/AKT/mTOR and p53, to induce biological changes in cancer cells [15–18], which are detailed in the following sections. Thus, it is not surprising that the role of EpCAM in cancers is highly context dependent. For example, enhanced EpCAM expression promoted invasion by preventing cell–cell adhesion and promoting immune escape, as well as activating downstream oncogenic genes in leukemia and colon cancer [13, 19]. In early esophageal cancer, decreased expression of EpCAM was found to induce EMT, thus promoting metastasis [20]. Given its versatile functions in cancer biology, EpCAM is considered as an attractive target for translational medicine. This molecule is taken as a biomarker for detecting CTCs and CSCs, providing potential new diagnostic and prognostic approaches [21, 22]. In addition, therapeutic methods targeting EpCAM have shown potential for cancer treatment [23, 24].

In this review, we systematically summarize the pathological functions of EpCAM and its crosstalk with other signaling pathways in cancers, as well as its potential role in cancer diagnosis or prognosis. Moreover, therapeutic approaches taking advantage of EpCAM are also introduced to shed light on the translational prospects of EpCAM, from the laboratory bench to the clinical bedside.

## Overview of EpCAM

### Basic structure

**Fig. 1:**
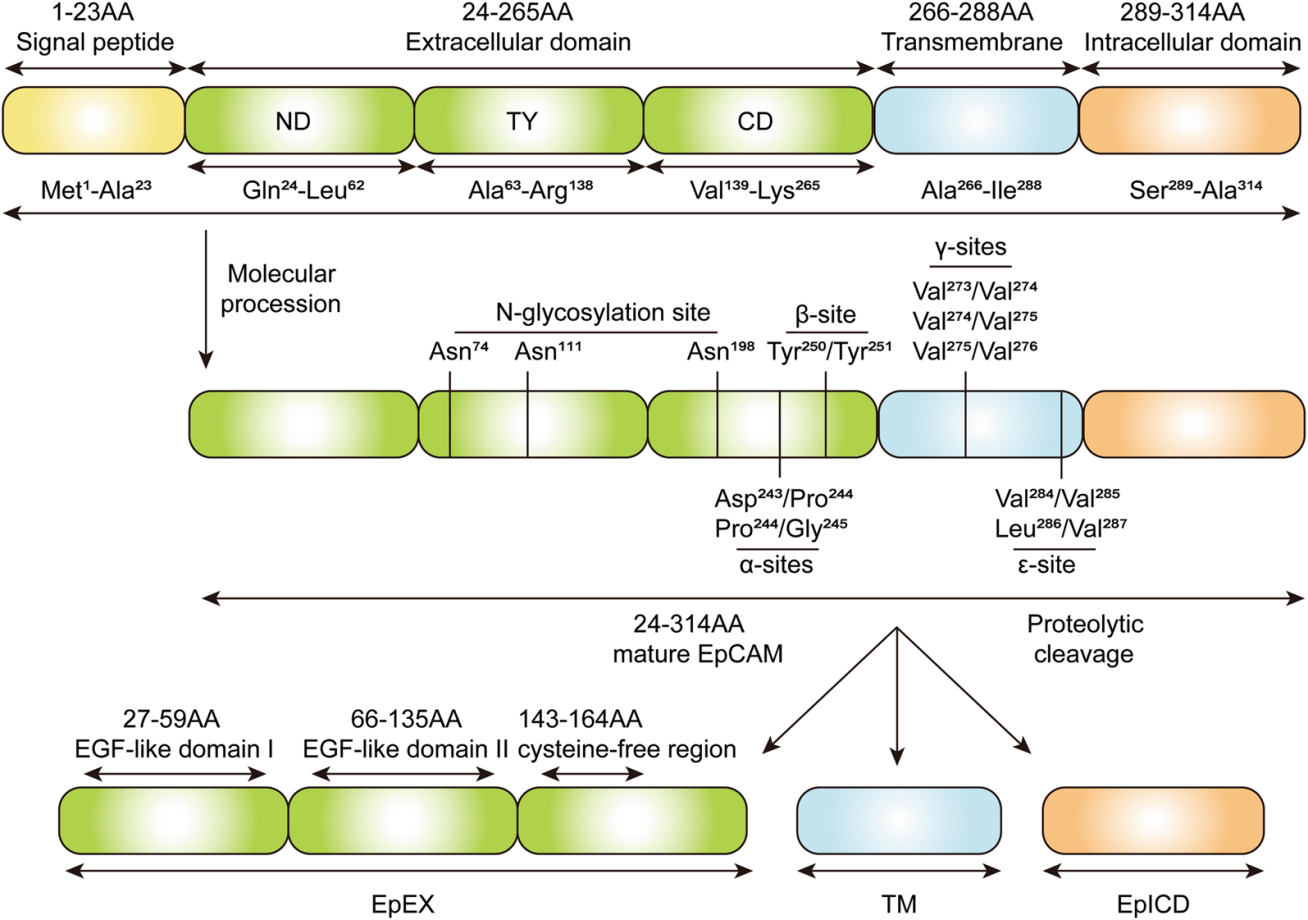
Schematic illustration of EpCAM structure. The premature EpCAM molecule is comprised of 314 amino acids (AA), including a signal peptide, which is cleaved during maturation. The mature membrane-bound EpCAM protein consists of an extracellular domain (EpEX), a transmembrane domain (TM) and an intracellular domain (EpICD). EpEX contains an N-terminal domain (ND), thyroglobulin-domain (TY) and C-terminal domain (CD), including three N-glycosylation sites. Four cleavage sites (α, β, γ, ε-sites) locate on EpCAM molecule, in which can be cleaved into soluble EpEX, TM and free EpICD. Human EpCAM contains two α-sites for ADAM protases, a β-site for BACE1 cleavage, and γ-secretase mediated cleavage on γ-sites and ε-sites

EpCAM is a single-chain membrane-spanning protein consisting of three major domains, termed EpEX, transmembrane domain (TM) and EpICD (Fig. 1). EpEX is consist of a cysteine disulfide-bonding N-terminal domain (ND), a thyroglobulin-type A1 (TY) domain formed by a cysteine-rich motif, and a C-terminal domain (CD) [25]. EpEX contains two epidermal growth factor (EGF)-like repeats, followed by a cysteine-free region (Fig. 1) [26]. Consistent with EGF-like peptide in the EpEX domain, signaling mediated by the EpEX is involved in EGFR-mediated signaling pathways, which is specified in the following section [17].

The transmembrane domain of EpCAM is a highly conserved valine-rich and leucine-poor helix [25]. Pavsic et al. demonstrated that the two neighboring TMs located close to each other and the cis-oriented subunits of TM helices enhance the stability of the EpCAM cis-dimer [25]. In addition, EpCAM-induced ERK and myosin downregulation was blocked in TM mutant Madin-Darby canine kidney cells, indicating its involvement in signaling transduction [27].

EpCAM can generate a membrane-tethered C-terminal fragment (EpCTF) by proteolytic cleavage. The EpCTF molecule is subsequently processed by γ-secretase into EpICD, a short tail of 26 amino acids [28].

### Epigenetic and posttranslational modifications of EpCAM

Early investigation unveiled that intact p53 could maintain EpCAM DNA methylation and inhibit the gene amplification [29]. Previous study indicated low expression of EpCAM suggested more invasive phenotypes. In this subgroup, EpCAM gene was shown abundant with hypermethylated histone 3 lysine 9. Treating with demethylating agents or histone deacetylase inhibitor could increase EpCAM expression and inhibit invasiveness [30]. In cells with hepatitis B virus X protein (HBx) expression, downregulation of suppressor of SUZ12 and ZNF198 induced histone modifications of the EpCAM promoter. Similar results were found in tumors derived from X/c-Myc transgenic mice. In addition, activation of DNA demethylation increased EpCAM expression and other acquisition of oncogenic processes [31].

The effects of protein glycosylation on cell proliferation, invasion, adhesion and signaling transduction have been reported in numerous studies [32]. In malignant tumors, such as breast cancer (BC), EpCAM was found to be hyperglycosylated [33]. N-Glycosylation mutation of EpCAM significantly impaired the EMT process in BC, thus inhibiting invasion and metastasis [14]. In addition, proliferation and apoptosis were correlated with the state of glycosylation in BC [10]. Proliferating human mammary epithelial cells (HMECs) express predominantly glycosylated EpCAM isoforms, while in confluent and contact-inhibited situations, the glycosylation of EpCAM is abolished [34]. Asn74, Asn111 and Asn198 were identified as three N-linked glycosylation sites of EpCAM (Fig. 1). The glycosylation of Asn198 is crucial for protein stability, with mutants Asn198 for Ala showing decreased overall expression and half-life of the molecule on the cell surface [33].

### Cleavage of EpCAM

**Fig. 2:**
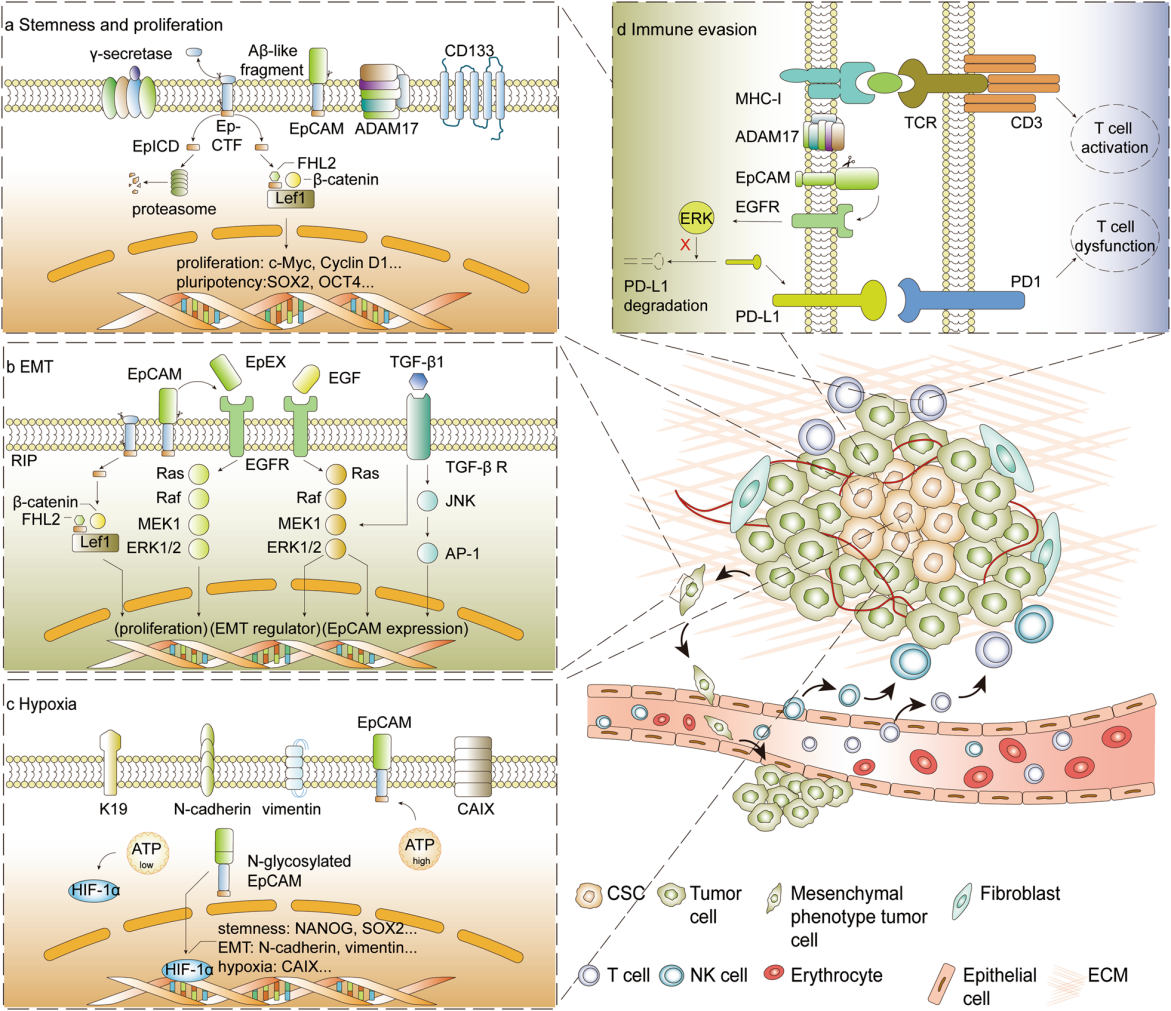
Roles of EpCAM in cancer development and progression. **a** EpCAM on CSCs membrane surface was cleaved by ADAM17 and γ-secretase, generating EpICD. Most of the EpICD is degraded by proteasome, while the remaining EpICD can bind with FHL2, β-catenin and Lef-1, forming the trans-nuclear complex to activate proliferation and pluripotency related genes. **b** EpEX/EGFR pathway and EpICD trans-nuclear complex can promote cell proliferation. EGF and TGF-β pathway can regulate EMT markers and EpCAM expression. **c** In hypoxic condition, EpCAM is upregulated in ATP-high state, whereas in ATP-low situation, HIF-1α is upregulated. CAIX is overexpressed mediated by HIF-1α. CAIX^+^, together with higher EpCAM and K19 expression HCC subgroup exhibited with high resistance to chemoembolization. Additionally, N-glycosylated EpCAM can regulate HIF-1α and promote EMT and stemness related properties. **d** MHC-I/TCR interaction serves as T cell activation signals. While activated EpEX/EGFR/ERK pathway results in reduction of PD-L1 ubiquitination degradation. PD-L1 on tumor surface hampers activation of CD8^+^ T cells and leading to immune escape. *CAIX* carbonic anhydrase-IX, *ECM* extracellular matrix, *FHL2* four and a half LIM domain protein 2, *HIF-1α* hypoxia inducible factor 1α, *K19* keratin 19, *Lef-1* lymphoid enhancer factor 1

Membrane-bound EpCAM can be cleaved via regulated intramembrane proteolysis (RIP) (Fig. 2a), which is frequently activated by cell–cell contact or soluble EpEX in cancers [7, 35]. Additionally, hypoxia was also reported to promote RIP, which usually appears in the tumor microenvironment [11]. Four sites (α, β, γ, ε-sites) on the molecule were shown to be essential for proteolytic cleavage (Fig. 1) [36]. Sequential cleavage of EpCAM RIP initiates α-secretase (ADAM)- and β-secretase (BACE)-induced EpEX shedding, leaving the EpCTF fragment. Subsequently, γ-secretase catalyzes the EpCTF fragment and releases EpICD [28]. The shedding of EpEX promotes the release of EpICD and may be a prerequisite for the cleavage of the intracellular domain of EpCAM [7]. Additionally, ADAM and BACE1 cleavage varies among different species and pH conditions [37]. The process of γ-secretase proteolysis is rather slow and the majority of free EpICD is degraded by the proteasome [28].

In addition to RIP, EpCAM is also a substrate of matriptase, a membrane-anchored protease [38]. During cleavage, the short N-terminus of EpCAM remains attached to EpEX via a disulfide bond [39]. Moreover, the EpCAM monomer is relatively sensitive to matriptase proteolysis. Intact EpCAM-claudin stabilization is crucial for the homeostasis of intestinal epithelial cells, but the interaction is blocked by matriptase cleavage [39].

### EpCAM and Trop2

Trop2 is mostly studied in isolation, regardless of its high similarity with EpCAM. Proteolytic cleavage in ectodomain of Trop2 is mediated by ADAM17 and in intracellular domain by γ-secretase [40]. Apart from ADAM17, a recent study found that Trop2 could be cleaved by ADAM10 at Arg87-Thr88. Their reports indicated that cleavage at Arg87-Thr88 is mandatory for Trop2 induced tumor growth [41]. Analog to EpCAM, the ectodomain of Trop2, Trop2EX, forms a Trop2 dimer. Compared with EpCAM, the ND in Trop2 is more exposed. Moreover, the ADAM17 cleavage site in Trop2 dimer is on the exposed edge of a β-strand, which makes it possible for the proteolysis in a dimeric form [42]. Reports demonstrated that Trop2EX could modulate α5β1 integrin to promote prostate cancer metastasis [43]. The cytosolic tail, Trop2IC was also demonstrated to obtain stem-like properties via β-catenin [40]. Similar to EpCAM, Trop2 was reported to induced EMT mediated by β-catenin [44]. Apart from RIP, matriptase-mediated cleavage of EpCAM and Trop2 was shown to destabilize their interaction with claudins [38]. In fact, the redundancy of researches on EpCAM or Trop2 could inspire each other and may should be compiled to take a step forward the truth [2]. Comprehensive reviews of Trop2 can be refereed to Liu et al. [45] and Lenart et al. [46].

## Biological and physiological functions of EpCAM

### Puzzles of EpCAM in cell adhesion

The role of EpCAM in cell adhesion is puzzling and controversial, several models have been proposed to explain the involvement of EpCAM in cell adhesion [3]. The initial impression of EpCAM served as an adhesion molecule due to the phenomena that EpCAM transfected L cells, which lack classic cadherin mediate adhesion, frequently formed the cell–cell connections and mediated multicellular aggregation in suspension culture [47]. Later research demonstrated EpCAM cis-dimer and its anchor to actin cytoskeleton with α-actinin provided the potential for cell adhesion [26]. With deeper investigations on the structure of EpEX, TY domain of two neighboring EpCAM was found to form a cis-dimer which could be further enhanced by dimerization interface in TM domain. The dimeric form of EpEX provided references for the hypothesis of trans-tetrameric intercellular unit [25]. Recent reports, however, almost refuted the proposed tetramer for homophilic adhesion. Results indicated that EpEX cis-dimer was highly predominant. The following results further dismissed the possible tetramer model considering its functions and avidity nor native-like orientation [48].

Contrarily, in epithelial cells, EpCAM was found to impair E-cadherin-mediated cell adhesion and overall strength of intercellular adhesion [49]. Later research indicated that EpCAM hampered E-cadherin-mediated adhesion by interfering the α-catenin linkage with F-actin [50]. At the same time, Maghzal et al. demonstrated that EpCAM buffered actomyosin contraction by inhibition of PKC and provided proper cortical tension, which may contribute to non-specific cell–cell adhesion [3]. Another possibility is that EpCAM is a heterophilic cell adhesion molecule with partner still unclear [2].

### EpCAM in epithelial integrity and non-malignant diseases

As demonstrated in wild type mice, EpCAM was abundant in developing intestinal, whereas for EpCAM knockout mice, the tight junctions (TJs) structure was abnormal, companied with reduction of claudins, especially claudin-7 [51]. Later investigation found that downregulated EpCAM was correlated with decreased overall level of claudin-1 and claudin-7, but their accumulation in TJs was increased, which suggested EpCAM could recruit and distribute claudins for TJs [52]. Previous report has indicated that mutation of EpCAM was found responsible for congenital turfing enteropathy (CTE), a lethal infancy diarrhea characterized by villus atrophy [53]. Cleavage of EpCAM by matriptase at Arg80 was demonstrated to destabilize EpCAM and claudin-7 interaction, leading to lysosomal degradation of claudin-7. This finding explained the role of EpCAM in intestinal epithelium homeostasis [39]. In addition, for CTE patients, absence of EpCAM was suggested to disrupt the polarity of choanocytes and lead to ductopenia [54]. Apart from CTE, a previous study suggested that intestinal epithelial cells derived extracellular vesicles (EVs) could alleviate inflammatory bowel disease by targeting CD4^+^ T cell and dendritic cells. Whereas the EVs required EpCAM to localize to gastrointestinal tract [55]. Similar to intestinal epithelial cells, EpCAM were found to regulate claudins in keratinocytes, the constituent of the skin barrier. Matriptase and its cognate inhibitor, hepatocyte growth factor activator inhibitor proteins, regulated EpCAM cleavage and might offer a novel perspective for matriptase dysregulation-induced ichthyosis [38]. Due to the pivotal biological function, the side effects of therapeutic approaches targeting EpCAM must be taken into consideration.

## Crosstalk between EpCAM and other signaling pathways during cancer progression

### Transforming growth factor β (TGF-β)

In HBV-infected hepatic progenitor cells, the expression of TGF-β1 and HBx was positively correlated with EpCAM and CD90. TGF-β1 was found to cooperate with HBx to enhance the transcription of miR-199a-3p by activating the JNK/c-Jun signaling pathway, which further elevated the expression of stemness markers, including EpCAM [16]. For breast cancer, TGF-β1 was found to promote EMT by upregulating EpCAM via JNK/AP-1 activation [56] (Fig. 2b). However, during the TGF-β1-induced EMT of esophageal cancer cells, EpCAM expression on the cell surface was substantially reduced [20]. In MCF-10A, A549 and HaCaT cell lines, TGF-β1 was found to downregulate the level of EpCAM, and knocking down EpCAM enhanced TGF-β1-induced EMT [57].

Conversely, TGF-β1 treatment of HMECs with ectopic expression of EpCAM resulted in additional small, strongly light-refracting cells and fewer cells positive for senescence-associated β-galactosidase. These observations demonstrated that EpCAM acted against the induction of growth arrest by TGF-β1 [34]. Pal et al. reported that the TGF-β and cyclin D1 (CCND1) signaling pathways were significantly activated in EpCAM^+^/CD45^−^ metastatic castration-resistant prostate cancer [58]. In non-small cell lung cancer (NSCLC) cell lines, the expression of E-cadherin, EpCAM and αvβ6 was found to induce the transcription of TGF-β1 and TGF-βR1 in normal fibroblasts to modulate tumor progression and therapeutic responsiveness [59].

### Wnt/β-catenin

EpCAM exerts its functions by mediating Wnt/β-catenin signaling in multiple cancers. Following RIP processing, shed EpICD forms a transcriptional complex with four and a half LIM domain protein 2 (FHL2), lymphoid enhancer factor 1 (Lef-1) and β-catenin to induce oncogenic transcription (Fig. 2a) [7]. Overexpression of EpICD contributed to elevated β-catenin, c-Myc and CCND1 levels, which were closely correlated with Wnt/β-catenin signaling [9]. In colon cancer, EpICD was found to interact with β-catenin and hypoxia inducible factor 1α (HIF-1α) to promote the transcriptional activity of HIF-1α-targeted genes (Fig. 2c) [19]. Downregulation of the transmembrane heparan sulfate proteoglycan syndecan-1 increased the expression of cell stemness markers via the activation of β1-integrins, focal adhesion kinase, and Wnt signaling [60]. In EpCAM^+^ hepatocellular carcinoma (HCC), as well as advanced cirrhosis liver tissues, autocrine Wnt signaling was activated, as evidenced by elevated expression of Wnt3, β-catenin, c-Myc and CCND1 [61].

Reciprocally, Wnt/β-catenin signaling is one of the most significant pathways for modulating EpCAM expression. In HCC, the β-catenin/TCF4 complex was found to regulate EpCAM transcription, promoting the self-renewal of liver CSCs [22]. Downregulation of GSK-3β, a Wnt/β-catenin inhibitor, increased the EpCAM^+^ subpopulation of cancer cells [62]. Decreased expression of βII-spectrin downregulated the expression of kallistatin, thereby activating Wnt/β-catenin signaling and increasing the EpCAM^+^ subpopulation of cancer cells [63]. Downregulation of cytokeratin 18 increased EpCAM expression by activating Wnt/β-catenin, leading to partial EMT and elevated stemness of breast cancer cells [64].

### PI3K-AKT

Previous studies showed that EpCAM^+^/CD45^+^ ovarian cancer acquired a more aggressive and drug-resistant phenotype by upregulating downstream effectors of PI3K/AKT signaling [65]. Knockdown of EpCAM in prostate cancer cell lines inactivated the PI3K/AKT/mTOR signaling pathway, thus sensitizing the cancer cells to chemo/radio-therapy [66]. In nasopharyngeal carcinoma (NPC), enhanced EpCAM expression downregulated PTEN while promoting the phosphorylation of AKT, mTOR, p70S6K and 4EBP1. Correspondingly, administration of the AKT inhibitor MK2206 or rapamycin impaired the invasion and stem-like phenotype of NPC cells by blocking the effects of EpCAM [15]. Additionally, a study demonstrated that deglycosylation of EpCAM in BC promoted the process of autophagy by activating the PI3K/Akt/mTOR pathway, thus resulting in a decreased proliferation rate [10].

### EGF/EGFR/ERK signaling

In human epithelial ovarian cancers, EGF was found to hinder cell migration by upregulating EpCAM and activating ERK1/2 signaling [67]. At the same time, activated ERK2 signaling suppressed EpCAM transcription in normal epithelial and multiple malignant epithelial cell lines by directly binding to the consensus ERK2-binding site in the EpCAM promoter, as well as indirectly by upregulating the EMT-related transcription factors SNAI1/2, TWIST1 and ZEB1, which bind to E-box sites in the promoter region. Conversely, depletion of EpCAM could enhance EGF-induced EMT [57].

In colon cancer, EpEX binds to EGFR and activates EGFR/ERK1/2/AKT signaling, which further promotes the RIP process [19]. In head and neck squamous carcinomas (HNSCCs), EpEX functions as a ligand of EGFR, whose binding leads to the phosphorylation of ERK1/2 and AKT, leading to EGFR-dependent proliferation. HNSCC cells treated with EpEX exhibited a dose-dependent repression of EGF-induced EMT-associated gene transcription. Therefore, the reports suggested that EpCAM impairs EGF-induced EMT by competitively binding with EGFR [5] (Fig. 2b), which is further specified in following section.

### P53

EpCAM-expressing stem-like mouse ovary cells developed into tumor-initiating cells following retrovirus-mediated transfection with c-Myc and K-Ras oncogenes and p53 depletion. Moreover, depletion of p53 increased the subpopulation of EpCAM^+^ primary breast cancer cells and led to enhanced tumorigenesis [68]. Sankpal et al. reported a dose-dependent decrease in EpCAM following the induction of p53, which repressed EpCAM expression by binding to its promoter region. Transcriptional repression of EpCAM contributes to the p53-mediated inhibition of breast cancer aggressiveness [69]. In primary human mammary epithelial cells, overexpression of EpCAM upregulated p27KIP1 and p53 in a posttranscriptional manner [34]. In mouse embryonic fibroblasts, ectopic expression of EpCAM or EpICD led to a significant decrease in p53 RNA levels and phosphorylated p53 protein [70]. In addition, EpCAM and p53 exerted their functions exclusively. In HCC, miR-30e-3p was found to play a tumor-suppressive role via a miR30e-3p/P53/MDM2 feedforward loop. Moreover, miR-30e-3p enhanced sorafenib resistance by targeting PTEN, p27 and EpCAM in cells lacking functional p53 [18].

### Other signaling pathways

In NSCLC, gemcitabine was found to decrease EpCAM expression by inactivating the HGF/cMET pathway [71]. p-21 activated kinase 4 (PAK4) was found to enhance stem cell-like characteristics through STAT3 signaling and increase the expression of EpCAM in pancreatic cancer cells. At the same time, PAK4 exhibited higher expression levels in triple-positive (CD24^+^/CD44^+^/EpCAM^+^) pancreatic CSCs than in triple-negative CSCs [72]. As previously stated, hypoxia was found to increase the N-glycosylation of EpCAM in BC, which induced the nuclear translocation of NF-κB, promoting EMT and stemness [11]. For EpCAM-dependent breast cancer invasion, the transcription factor AP-1 was reported to participate in the MEKK1/MKK7/JNK pathway, while EpCAM was shown to modulate the effects of NF-κB and IL-8 during BC progression [73].

### Noncoding RNAs (miRNAs, lncRNAs, circRNAs)

Recently, noncoding RNAs have emerged as pivotal players that regulate gene expression and signaling pathways to modulate cancer progression. In HCC, miR-30e-3p was found to bind the 3′UTR of EpCAM mRNA to downregulate its expression, thus inhibiting the stemness and chemoresistance of cancer cells [18]. Downregulated miR‐26b‐5p led to increased EpCAM transcription and a population of EpCAM^+^ HCC cells, as well as the overexpression of NANOG, OCT4, and SOX2, the direct targets of EpCAM [74].

Additionally, lncRNAs participate in the regulation of EpCAM expression. Lnc-TCF7 was demonstrated to upregulate EpCAM expression by binding and decreasing the abundance of miR-200c in a competitive endogenous RNA manner in glioma cells [75]. Lnc-DILC expression was found to be suppressed in EpCAM^+^ liver CSCs, and correspondingly, the EpCAM^+^ liver CSC subpopulation was increased following lnc-DILC depletion. Mechanistically, lnc-DILC suppresses autocrine IL-6/STAT3 signaling by binding to the promoter region of IL-6 and inhibiting the crosstalk between TNF-α/NF-κB signaling and the autocrine IL-6/STAT3 cascade in the EpCAM^+^ subpopulation [76]. High levels of lnc-THOR were observed in EpCAM^+^ liver CSCs. This increased the population of liver CSCs by stabilizing the mRNA of β-catenin, thus leading to increased chemoresistance [77]. In addition, 11 up- and 12 down-regulated circRNAs were identified in the livers of EpCAM^−/−^ mice compared with wild-type mice, implying the potential involvement of circRNAs in the regulation and activity of EpCAM signaling [78].

## Roles of EpCAM in tumor biology

### Cancer stemness

A previous study demonstrated that EpCAM is widely expressed on human embryonic stem cells and plays a pivotal role in differentiation [79]. Kuan et al. reported that EpEX and EpCAM could enhance the reprogramming efficiency of OCT4, SOX2, KLF4, as well as c-MYC, and induce fibroblasts into pluripotent stem cells through STAT and HIF-2α [80].

For cancers, EpCAM is a well-regarded marker of CSCs. Tumor cells expressing EpCAM exhibited an enhanced capacity to initiate cancer formation, as well as other stemness properties (chemo/radioresistance, elevated tumorigenicity, angiogenesis, hypoxia tolerance and metastatic colony formation ability) [81]. In HCC, the EpCAM^+^/AFP^+^ subgroup displayed self-renewal and differentiation abilities [62]. EpCAM^+^ leukemia and prostate cancer cells exhibited increased resistance to chemotherapies, and knocking down EpCAM sensitized the chemotherapeutic agent-induced cell apoptosis [13, 82]. In line with these findings, elevated EpCAM expression in breast CSCs exhibited increased resistance to radiotherapy and a more aggressive metastatic phenotype [83]. In addition, after doxorubicin (DOX) treatment, the proportion of EpCAM^+^/CD133^+^ was significantly increased in the EpCAM^−^/CD133^−^ nonstem HCC population, accompanied by more stemness properties and elevated tumor-forming ability [84]. Recently, one possible mechanism suggested that EpCAM could regulate the Nrf2/ARE (antioxidant response elements) pathway to acquire chemoresistance and reduce reactive oxygen species activity. Based on their investigation, the EpCAM/Nrf2 pathway might contribute to CSC features and chemoresistance [85].

### Cell proliferation promotion and cell death resistance

The presence of EpCAM^+^ cells indicated stronger growth and colony formation abilities in BC and HNSCC [85, 86]. In HCC, EpCAM^+^ and CD90^+^ cells were found to have distinct localization. CD90^+^ CSCs are an indicator of higher metastatic capacity, while EpCAM^+^ CSCs are associated with rapid growth [87]. In addition, nuclear EpICD accumulation was implied to be correlated with HCC cell proliferation [88]. The transcription of cell proliferation-associated genes (CCND1, Rb, Ki67, etc.) is induced by a nuclear complex consisting of EpICD together with FHL2 and the Wnt pathway components β-catenin and Lef-1 [7, 9]. The expression of EpCAM was found to be correlated with Ki67, CCND1 and phosphorylated Rb in many types of cancer [9, 35]. In addition, in lung cancer cells, reports suggested that SOX2 induced EpCAM/p21/cyclin A2 by binding to the EpCAM promoter, leading to enhanced cell proliferation. SOX9 attenuated the expression level of SOX2, resulting in reduced proliferation ability and a more invasive phenotype [89].

EpCAM is also involved in cell death modulation. Genome-wide RNA interference screening suggested that mitochondrial processing peptidase subunit beta (PMPCB) ensured sustainable development for EpCAM^+^ HCC. Blockade of PMPCB suppressed EpCAM expression and Wnt/β-catenin signaling, thus resulting in apoptosis of EpCAM^+^ HCC cells and tumor suppression [90]. A recent study revealed that deglycosylated EpCAM inhibited proliferation but activated the process of autophagy and apoptosis in BC [10].

### Cell metabolism and angiogenesis

In HCC, a subgroup of cells positive for carbonic anhydrase IX (CAIX), a hypoxia-related marker, was positively related to the expression of EpCAM and keratin 19 (K19). Additionally, EpCAM^+^/K19^+^ HCC cells under hypoxia showed resistance to arterial embolization therapy, resulting in poorer outcomes [91]. A distinct ATP state is associated with different levels of EpCAM and phenotypes. ATP-low MDA-MB-231 BC cells showed a higher level of HIF-1α, whereas EpCAM was found to be upregulated, together with a more invasive and metastatic potential in the ATP-high subgroup [92]. Furthermore, EpCAM was demonstrated to act as a regulator via the NF-κB pathway to modulate HIF-1α-dependent stemness and EMT properties [11] (Fig. 2c).

Notably, CSCs display flexible energy metabolism, ranging from mitochondrial oxidative phosphorylation (OXPHOS) to glycolysis under oxidative or hypoxic conditions [81]. EpCAM^+^ HCC exhibited more active expression of lipid metabolism-related genes [93]. Similarly, fatty acids, as well as their synthesis genes, were found to be significantly increased in EpCAM^+^ cells isolated from colon cancer patients [94]. Dichloroacetate was recently found to be able to divert metabolism toward OXPHOS, which downregulated the CSC markers CD24/CD44/EpCAM. More importantly, dichloroacetate inhibited the formation and viability of EpCAM^+^ pancreatic cancer cells [95]. Additionally, a recent study implied that EpCAM affected glycogen synthesis, which was associated with diseases of glycogen storage and might participate in liver-related diseases [78].

A comparison of four subgroups of HCC with distinct expression of EpCAM and AFP indicated that the EpCAM^+/^AFP^+^ population exhibited higher microvessel density and higher expression levels of vascular epithelial growth factor [12]. Furthermore, Sankpal et al. reported that EpCAM could modulate IL-8 and NF-κB transcription factor activity in BC invasion and angiogenesis [73]. Enhanced effectiveness by bispecific antibody targeting VEGFR2/EpCAM and its modulation of angiogenesis by decreasing IL-8 and IL-6 implied that EpCAM may be a promising target in preventing angiogenesis in cancer progression [96].

### Immune evasion and modulation

EpCAM was found to be involved in tumor immune modulation. Park et al. concluded that EpCAM-high HCC resisted natural killer (NK) cell-mediated cytotoxicity by upregulating carcinoembryonic antigen-related cell adhesion molecule 1 [97]. Similarly, EpCAM^+^CD45^+^ ovarian cancer cells escaped NK-cell-mediated immune surveillance by overexpressing major histocompatibility complex class I antigen (MHC-I) [65]. Additionally, NK cells were implied to promote HCC in HBV transgenic mice. Later, experiments on HBV transgenic mice revealed that NK-cell-derived IFN-γ promoted HCC via the EpCAM/EMT axis [98]. In leukemia, EpCAM was found to hinder immune surveillance by activating Wnt5B signaling. Accordingly, inhibition of EpCAM could enhance the sensitivity of tumor cells to immune surveillance [13]. Moreover, in xenograft mouse models, the protective environment for leukemia in the bone marrow could be counteracted by EpCAM antibodies. In vivo experiments showed that EpCAM blockade could enhance macrophage infiltration to efficiently eliminate tumor cells [13].

PD-L1 is considered a major aspect of the failure of immune surveillance by CD8^+^ T-cell-mediated cytotoxicity [99]. EGF/EGFR signaling triggered the release of EpEX from cell-surface EpCAM, after which the shed EpICD bound with EGFR to stabilize PD-L1 through the EGFR/ERK pathway in cancer cells (Fig. 2d) [6]. EpCAM and PD-L1 were also found to be upregulated in CD4^+^ T cells derived from colon cancer patients, which was regarded as an ineffective immune response. Further analysis indicated that p38/MAPK might be a potential target for EpCAM^+/^CD4^+^ T-cell-rich colon cancer patients [100]. Moreover, EpCAM antibodies succeeded in downregulating the PD-L1 level and enhanced the therapeutic efficacy of atezolizumab [6].

### Dynamic expression and context-dependent roles during EMT

EpCAM plays a dynamic and context-dependent role in EMT (Fig. 2d). As an adhesive molecule, the expression of EpCAM was speculated to be downregulated to increase the mobility of cancer cells [20]. However, EpCAM can reduce E-cadherin-mediated cell–cell adhesion by interfering with the link between α-catenin and F-actin [50]. Furthermore, EpCAM is suggested to be positively correlated with the expression of EMT-related genes, including N-cadherin, snail and vimentin [11, 87]. Indeed, inconsistent results were observed in different studies. During TGF-β1-induced EMT in esophageal cancer cells, EpCAM expression on the surface of the cell membrane was substantially reduced, together with enhanced migration, invasion and dissemination ability [20]. Similarly, low EpCAM expression enhanced EMT of cancer cells and was correlated with advanced tumor stage and lymph node metastasis in endometrial carcinoma [101]. However, EpCAM expression was elevated in TGF-β1-treated MCF-7 BC cells, which was demonstrated to promote EMT and cell migration [56].

**Fig. 3:**
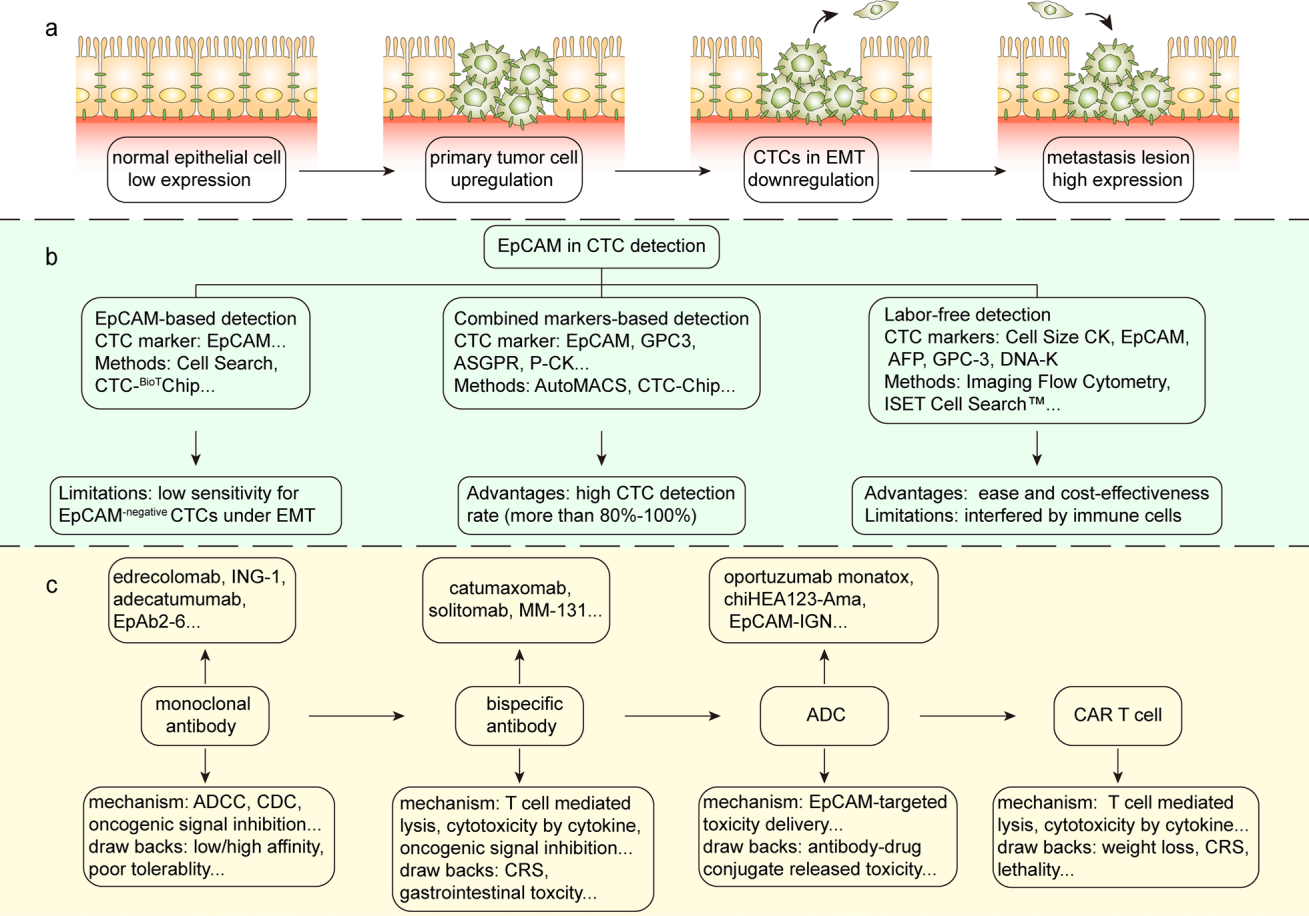
Changes and developments of EpCAM in metastasis, detection and immunotherapy. **a** Dynamic expression of EpCAM in EMT process of cancer cells. **b** EpCAM related CTC detection methods, advantages and their limitations. **c** Development of EpCAM mediated immunotherapies. *ADC* antibody–drug conjugate, *ADCC* antibody-dependent cell-mediated cytotoxicity, *CDC* complement-dependent cytotoxicity, *CRS* cytokine release syndrome

A hypothesis was proposed to integrate the inconsistent observations in the cancer EMT process. Driemel et al. considered the expression of EpCAM to be dynamic rather than consistent. EpCAM was speculated to have low expression in normal epithelial cells for tissue integrity. Elevated expression in primary tumor formation might participate in proliferation and adhesion. During EMT or dissemination, downregulation of EpCAM was considered to be associated with migration and invasion. Finally, re-expression in distant micrometastatic lesions contributed to proliferation signals [20] (Fig. 3a).

Another interesting point is that the role of EpCAM is context dependent. EGF/EGFR signaling promoted HNSCCs proliferation and migration. EMT induced by EGF treatment was completely blocked by ERK inhibitor (Cetuximab, Erlotinib, and AZD6244) but not by AKT-inhibitor MK2206 in HNSCCs. And following EGF treatment, FaDu (EMT-responsive) cells induced a rapid and strong activation of ERK1/2, whereas Cal27 cells (EMT-nonresponsive) were only moderately and more transiently activated. These results implied the dominant role of ERK1/2 in EGF-mediated EMT. However, as the competitor of EGF to EGFR, EpEX was demonstrated to activate ERK to a moderate level. Only high dose of EpEX could promote cell proliferation and counteracted EGF-induced migration. Consistently, EpCAM^high^/EGF^low^ HNSCCs patients presented with proliferative phenotypes, while EpCAM^low^/EGF^high^ of carcinomas exhibited enhanced EMT abilities and poor survival [5]. Additionally, EGF treatment was indicated to trigger cleavage of membrane EpCAM. Later, the EpICD translocated complex could regulate cell mobility-related genes and exhibit EMT properties [102]. Sankpal et al. concluded that when ERK activation dominates invasiveness during EMT, upregulated EpCAM expression may counteracts pro-metastatic ERK signaling. Correspondingly, when activated ERK signaling does not contribute to a more aggressive phenotype, nuclear localization of EpICD, which also potentiates cell growth and EMT, as well as other mechanisms, may be the predominant drivers of cancer progression [57].

## Applications of EpCAM in clinical oncology

### The expression pattern and prognostic value of EpCAM in cancers

Given its versatile functions in cancers, EpCAM participates in multiple steps of cancer progression. In addition to its diagnostic and prognostic value for the prediction of metastasis and the detection of CTCs, its unique expression patterns in cancers potentiate it as a valuable biomarker for diagnosis and prognosis, which are detailed in Table 1.

**Table 1:** The expression pattern and clinical significance of EpCAM in cancers

Cancer type	Expression	Related phenotypes	Clinical characteristics	Mechanism/pathway	Ref.
Hepatocellular carcinoma (HCC)	Upregulated	Stemness	Progenitor features with poor prognosis	Wnt/β-catenin signaling	[62]
	Downregulated	Chemo-resistance	resistant to cisplatin	Hedgehog signaling	[154]
Nasopharyngeal carcinoma (NPC)	Upregulated	EMT and stemness;	Associated with metastasis and shorter survival	PTEN/AKT/mTOR signaling	[15]
Thyroid cancer	Upregulated	–	Shorter OS	Wnt/β-catenin signaling	[155]
Colon cancer	Upregulated	Cell migration, proliferation, tumor growth, hypoxic adaption	Poor prognosis and metastasis	EpEX/EGFR/ERK1/2 and Wnt/β-catenin signaling; Hypoxia-related gene expression	[7, 19]
Breast cancer	Upregulated	Cell stemness, invasion, EMT,	Shorter DFS and OS	Wnt/β-catenin, JNK/AP-1, NF‐κB signaling	[11, 86, 156]
	Downregulated	EMT	Invasive phenotype and poor prognosis	EpEX, EGF, and TGFβ1 signaling with ERK	[57]
Leukemia	Upregulated	Chemo-resistant, Immune evasion, stemness	Chemo-resistance and relapse	Wnt5B signaling	[13]
Esophageal adenocarcinoma (EAC)	Upregulated	Cell cycle, proliferation, migration	Poor prognosis and lymph node metastases, association with Barrett’s esophagus and EAC progression	Wnt/β-catenin signaling, CCND1 regulation	[20, 157]
	Downregulated	EMT, cell migration, invasion and dissemination	Association with CTCs	–	[20]
HNSCC	Upregulated	Cell cycle progression	Correlation with proliferation marker	Regulation on CCND1 and Rb	[9]
	Downregulated	EMT	Poor prognosis	EpEX/EGFR/ERK1/2 signaling	[5]
NSCLC	Upregulated	EMT, metastasis, invasion	Poor prognosis and CTCs	HGF/cMET and αvβ6/TGF-β signaling	[59, 71]
Ovarian cancer	Upregulated	Chemo-resistance, invasion, immune evasion	Chemo-resistance, metastasis, immune evasion	PI3K/AKT signaling, MHC-I overexpression	[65]
Ovarian cancer	Downregulated	Low response rate to chemo-therapy	Poor OS and tumor grade	–	[158]
Pancreatic cancer	Upregulated	Stemness, Chemo-resistance	Recurrence and chemo-resistance	PAK4/STAT3 signaling	[72]
Prostate cancer	Upregulated	EMT, proliferation, chemo/radio-resistance	Lymph node metastases, chemo/radio-resistance	PI3K/AKT/mTOR signaling	[66, 82]
Endometrial carcinoma	Downregulated	EMT, cell invasion	Advanced tumor stage and lymph node metastasis, poor survival	Erα/EpCAM signaling axis	[101]
	Upregulated	–	Poor prognosis	EGF/EpCAM/BMP signaling	[159]
Gastric cancer	Upregulated	Cell proliferation, migration	Poor OS, lymph node metastasis	Interaction with E-cadherin, β-catenin, PS-2, and ADAM17	[160, 161]
Renal cell carcinoma	Upregulated	Reduced migration, tumor necrosis	Better prognosis and OS and DSS	–	[162]

### EpCAM-based CTC detection and diagnostic methods

CTCs are considered as a promising diagnostic target for the detection of cancer metastasis [103]. EpCAM is proposed as a marker for detecting CTCs, given that it is rarely expressed on the surface of leukocytes but highly expressed in the majority of epithelium-derived cancers, such as BC, HCC and colorectal cancer (Fig. 3b) [104]. Since CTCs are defined as nucleated EpCAM^+^/CK^+^/CD45^−^ cells, EpCAM-based CTC detection, the CellSearch system has been applied for CTC detection [105]. Expanded coverage of cytokeratin or other indicators (e.g., vimentin) and EpCAM showed promising future in arterial and venous blood CTC monitoring and prognostic values [21, 104]. Nevertheless, the traditional detection of CTCs via EpCAM has its drawbacks. Conventional EpCAM-based CTC detection strategies were restricted to EpCAM-positive CTCs and carried the risk of overlooking the most aggressive HCC CTC subgroups because of EMT, resulting in an underestimate of the overall number of CTCs in circulation [106]. In fact, a recent study suggested that prostate CTCs cocultured with platelets showed decreased EpCAM expression levels but elevated platelet marker CD61, accompanied by a higher proliferation rate during EMT. The results indicated that CD61, rather than EpCAM, might be an alternative diagnostic marker in this situation [107].

Advances have been made with the aim of overcoming the nonnegligible false negative rate of CTC detection via EpCAM. Alternative markers have been applied for combined marker-based CTC detection. Investigation suggested that for CTCs not expressing EpCAM, CD44v6 detection is an alternative approach for the subgroup. That is, CD44v6 defined a new CTC population without EpCAM [108]. Similarly, the same report demonstrated that for urothelial cell carcinoma, EpCAM and urokinase plasminogen activator receptor are complementary targets in cancer diagnosis [109]. Recently, Court et al. demonstrated that with a multimarker panel of EpCAM, ASGPR, and GPC3, 97% of patients with HCC were CTC positive [110].

Furthermore, a comprehensive label-free approach was optimized for the detection of HCC-CTCs by phenotypic and karyotypic characterization. In addition, the quantity of polyploid (≥ pentasomy 8) EpCAM^+^ CTCs (cutoff: ≥ 1 cell), small EpCAM^−^ CTCs with trisomy 8 (≥ 5 cells), positive detection of circulating tumor microemboli (≥ 1), and increased triploid CTCs were linked with a poor prognosis in postoperative patients [111]. EpCAM-related and non-EpCAM methods have been universally applied in CTC detection [112].

### EpCAM in extracellular vesicles

As an intercellular communication medium, exosomes and microsomes have been shown to play pivotal roles during cancer pathogenesis by transferring bioactive substances [113]. A study revealed that serum-derived microsomes/exosomes in prostate cancer showed a four- to fivefold increase in the expression levels of EpCAM compared to healthy controls [114]. Quantitative analysis of EpCAM and other biomarkers on exosomes has shown prospects in blood sample identification of pancreatic and breast cancer [115]. Apart from carcinomas, for osteosarcoma-derived exosomes, analysis of EpCAM, CD63 and vimentin expression levels has exceeded traditional histological biopsy with high specificity and accuracy, as well as a low risk of tumor spread [116]. Furthermore, considering that EVs may fuse with the membranes of cancer cells, these biomarkers on EVs can be localized to cell membranes and serve as therapeutic targets. EpCAM and CD73 bispecific antibodies could selectively targeting EpCAM^+^ carcinoma-derived EVs. Moreover, enhanced efficacy to inhibit CD73 EVs-mediated immune suppression was found compared with targeting CD73 alone [117].

### Chemical modification and targeted treatment

Compared to conventional antibody-mediated tumor therapy, aptamers can be utilized to enhance the specificity and affinity of pharmacological reagent delivery. Since EpCAM is broadly expressed on cancer cells, especially CSCs, EpCAM-targeting aptamers are widely used in cancer treatment.

Conjugation of EpCAM-specific aptamers to DOX resulted in a high concentration and prolonged maintenance of DOX in the nuclei of EpCAM-expressing colorectal CSCs [118]. Furthermore, an EpCAM-aptamer-based platform for specific gene knockdown may be a novel therapeutic approach to overcome cancer resistance and immune evasion [23]. In human HER2^+^ and basal-A triple-negative BC, EpCAM aptamer-linked small-interfering RNA chimeras (AsiCs) enhanced the immune response against tumors that are insensitive to checkpoint blockade. AsiC mixtures were more effective than individual AsiCs and exhibited a synergistic effect with anti-PD-1 checkpoint inhibitors. Additionally, AsiC combinations could effectively induce the expression of neoantigens and cytokine production for cytotoxicity and increase tumor phagocytosis [23]. Similar to the bispecific antibody, simultaneously targeting CD44 and EpCAM with a bispecific aptamer showed higher efficacy than either aptamer alone. Moreover, the aptamer exhibited no toxicity to the host and no activation of innate immunity [119].

EpCAM aptamers also find their place in nanoplatforms for delivering therapeutics. Given the expression of CD47 and PD-L1 during tumorigenesis, designed EpCAM-targeted cationic liposomes containing si-CD47 and si-PD-L1 could target EpCAM-rich cancer cells to downregulate both CD47 and PD-L1 proteins. Moreover, these multiple nanoparticles exhibited high affinity for EpCAM^+^ tumor cells and low toxicity to healthy cells, resulting in enhanced immunotherapeutic efficacy [120]. In addition, liposomes based on HER-2 with anti-EpCAM toxin showed promise for BC treatment [121].

## Immunotherapeutic applications of EpCAM

### EpCAM monoclonal antibodies (mAbs)

Numerous promising EpCAM antibodies for cancer treatment have been reported in preclinical trials (Table 2). The first monoclonal EpCAM antibody, edrecolomab (17-1A), is an IgG2a derived from the ascites of murine. The antibody was applied in various adenocarcinomas and showed limited effects, which may should be blamed to its low affinity with tumors [122, 123]. The human-engineered and humanized antibodies, ING-1 and 3622W94, were then developed and tested in adenocarcinomas [123]. The two antibodies exhibited high affinity with EpCAM, but led to low tolerance and pancreatitis [124]. The full human IgG1 antibody, adecatumumab (MT201) exhibited dose-dependent anti-tumor activities and well-tolerance for metastatic breast cancer (MBC) [125]. For post-prostatectomy cancer patients with baseline PSA levels ≤ 1 ng/ml and high EpCAM-expressing, high dose adecatumumab treatment delayed disease progression [126]. A phase I study for hormone refractory prostate cancer patients and a phase IB study for EpCAM-positive relapsed or refractory advanced-stage breast cancer confirmed the feasibility of adecatumumab [127, 128]. EpAb2-6, a new EpCAM neutralizing antibody, exhibited antitumor effects via inhibiting the nuclear translocation of EpICD/β-catenin complexes and inducing apoptosis in colon cancer cells [19]. Moreover, EpAb2-6 impeded tumor progression by increasing high temperature requirement A2 expression and decreasing PD-L1 protein levels to enhance the cytotoxic activity of CD8^+^ T cells [6].

**Table 2:** Immunotherapeutic applications of EpCAM in cancer treatment

Name	Type/variate	Drawback	Application/mechanism	Ref.
Edrecolomab (17-A)	mAb	Murine IgG, low affinity	Phase II in various adenocarcinomas, phase II/III in NSCLC	[123, 163, 164]
ING-1	mAb	High affinity, pancreatitis	Phase I in various adenocarcinomas	[123]
3622W94	mAb	High affinity, pancreatitis	Phase I in various adenocarcinomas	[123]
Adecatumumab (MT201)	mAb	Limited effects on tumor regression	Phase II in MBC and prostate cancer	[125, 126]
EpAb2-6	mAb	–	In vitro for colon and lung cancers	[6, 19]
Catumaxomab (Removab)	BsAb	CRS	Malignant ascites, phase II/III in ovarian cancer and phase II in gastric cancer	[123, 129]
MM-131	BsAb	–	In vitro experiments for HCC, BC, gastric and lung cancers	[131]
MT110 (Solitomab)	BiTE	Gastrointestinal toxicity	Phase I in advanced solid tumors	[136]
1H8/CD3	BiTE	–	In vivo and in vitro experiments for HCC	[137]
EpCAM16	BiKE	–	In vitro experiments for BC, prostate, colorectal and head and neck cancers	[138]
1615EpCAM	TriKE		In vitro for various carcinomas	[137]
Tucotuzumab celmoleukin (EMD 273,066, huKS-IL2)	ADC	Lymphopenia and hypophosphatemia	Phase I/IB in solid cancers	[143, 145]
Oportuzumab monatox,	ADC	Pain at the injection site	Phase I/II in HNSCCs and bladder carcinomas	[142]
Citatuzumab bogatox	ADC	Fever, hypotension and hypoalbuminemia	Phase I in advanced epithelium tumors	[142]
ChiHEA125-Ama	ADC	ADC released toxicity	In vivo and in vitro experiments for pancreatic, colorectal, breast and bile duct cancers	[146]
EpCAM-IGN	ADC	–	In vivo and in vitro experiments for various cancers	[147]
CAR T cell	Rapamycin pretreatment	–	AML	[149]
	HsBCL9CT-24 cotreatment	–	Promoting infiltration and impeding exhaustion of T cells	[150]
	Bispecific CAR T cell	–	In vitro experiments for various carcinomas	[24]
CAR NK-92 cell	Regorafenib cotreatment	–	Promoting T cells infiltration and inhibiting immune suppressive cells	[152]

### EpCAM bispecific antibodies (BsAb) and variations

Bispecific or chimeric antibodies are developed to enhance anti-tumor efficacy (Table 2). Catumaxomab (Removab), with two antigen-binding sites (EpCAM and CD3) and a functional Fc domain, initiates T-cell-mediated lysis, cytokine-related cytotoxicity, antibody-dependent cell-mediated cytotoxicity (ADCC) and complement-dependent cytotoxicity (CDC) in vitro [129]. In patients with malignant ascites from a phase II/III study, the catumaxomab-treated group exhibited significantly longer overall survival (OS) [130]. MM-131, a bispecific anti-EpCAM/Met antibody, exhibited both HGF-dependent and -independent inhibition of Met signaling, inhibiting the proliferation of Met-driven HCC, BC, gastric cancer and lung cancer [131].

Bispecific T-cell engaging antibodies (BiTE) enhance the patient’s immune response to tumors by linking T cells to cancer cells, stimulating T-cell activation, tumor killing and cytokine production [132, 133]. EpCAM, as a common highly expressed molecule in cancer cells, is taken as a tumor-associated antigen. Solitomab (MT110), the bispecific T-cell engaging antibodies (BiTE) with two single chain binding to EpCAM and CD3, was effective against primary uterine and ovarian carcinosarcoma [134]. The mechanism of MT110 mainly relies on release of perforin and granzymes after T-cell activation [134, 135]. However, solitomab was finally abandoned due to its gastrointestinal toxicity [136]. Anti-EpCAM BiTE 1H8/CD3 succeed to inhibit the growth of HCC xenografts and reduced the expression of most CSC biomarkers [137]. To avoid harmful cytokine toxicity induced by activation of T cells, bispecific NK-cell engager (BiKE), targeting CD16 on NK cells and EpCAM on tumor cells is developed. It mediates ADCC against EpCAM^+^ HT-29 colon cancer cells [138]. Modified with interleukin-15 cross-liner, the trispecific NK-cell engager (TriKE) displays improved activation, proliferation and survival on NK cells compared with BiKE [139].

### EpCAM antibody–drug conjugates (ADCs)

EpCAM antibody–drug conjugates (ADCs) are also generated for EpCAM^+^ cancer therapies (Table 2). Oportuzumab monatox is comprised of an anti-EpCAM single-chain variable fragment and a fragment of pseudomonas exotoxin A (ETA), which displays anti-tumor effects after the ADC is internalized and ETA is released [140]. Similarly, citatuzumab bogatox is consist of anti-EpCAM Fab with non-immunogenic toxin of bougainvillea spectabilis [141]. Phase I and II clinical trials in various cancers showed their well tolerance and effectiveness for cancer treatment [142]. Tucotuzumab celmoleukin (EMD 273,066, huKS-IL2) is an immunocytokine by genetically fusing interleukin-2 (IL-2) to a humanized monoclonal antibody against EpCAM [143]. The novel antibody-based therapy may activate lymphocytes by IL-2. In addition, the combination of tucotuzumab celmoleukin with radiofrequency ablation enhanced anti-tumor effects and immunologic memory in murine colon cancer [144]. The safety and effectiveness of tucotuzumab celmoleukin for cancer patients were demonstrated in phase I and IB studies [143, 145]. Some more ADCs are emerging as promising EpCAM-targeting complexes for cancer treatment, evidenced by in vitro cell lines assays and in vivo animal experiments. ChiHEA125-Ama, conjugate of α-amanitin with a chimerized anti-EpCAM monoclonal antibody, exhibits tumor-suppressing potential in pancreatic, breast and various other cancers [146]. Correspondingly, attachment of indolinobenzodiazepine pseudodimers (IGN) to EpCAM (EpCAM-IGN) antibodies exhibited dose-dependent anti-tumor activity [147].

### Chimeric antigen receptor (CAR) T cells

Apart from antibody-mediated treatments, cellular immunity, especially T-cell therapy, shows prospects in cancer treatment. A recent study revealed that CD8^+^ T cells with ectopic expression of miR-200c and EpCAM exhibited reduced apoptosis and enhanced responses to both solid and liquid tumors. They suggested that the miR-200/EpCAM axis promoted the state for T cells to be poised for memory-like features [148].

In fact, EpCAM has already found its place in CAR T cells for cancer treatment (Table 2). EpCAM^+^ acute myeloid leukemia (AML) displays enhanced tumorigenicity and chemoresistance compared to EpCAM^−^ AML cells. Neither normal bone marrow cells nor peripheral blood mononuclear cells express EpCAM. Thus, CAR T targeting EpCAM stand out as an ideal method for treating AML. Moreover, rapamycin pretreatments can attenuate mTORC1 activity and upregulate CXCR4, which promotes the migration and penetration abilities of EpCAM CAR T cell for eliminating acute myeloid leukemia (AML) in bone marrow [149]. The expression level of EpCAM corelated with prognosis and metastasis of colorectal cancer, CAR T cells targeting EpCAM selectively destroy cancer cells with high EpCAM expression. Moreover, Wnt pathway inhibitor hsBCL9CT-24 has shown the potential to improve T cell infiltration, to promote effector T cells and to impede exhaustion of CAR T cells [150]. A current study demonstrated that bispecific CAR T cells targeting EpCAM and intercellular adhesion molecule 1 (ICAM-1) displayed superior efficacy in eradicating solid tumors. Moreover, activated CAR T cells against EpCAM result in upregulation of ICAM-1, leading the tumors to be more susceptible to bispecific CAR T cells [24].

Patients with CAR T cell therapies may suffer with cytokine release syndrome (CRS) and immune effector cell associated neurotoxicity syndrome, while CAR NK cell treatments have demonstrated the advantages in the unique biological attributes and safety [151]. CAR NK-92 cells therapy has demonstrated significant efficacy against human colorectal cancer. Cotreated with regorafenib improves EpCAM CAR NK-92 cells by enhancing immune cell infiltration, downregulating immune suppressive cell subsets and improving tumor vasculature [152]. Additionally, induced EpCAM CAR NK cells derived from modified induced pluripotent stem cells displays anti-tumor activities as well, which provides a novel approach to generate CAR NK cells more efficiently [153].

## Conclusions and perspectives

**Fig. 4:**
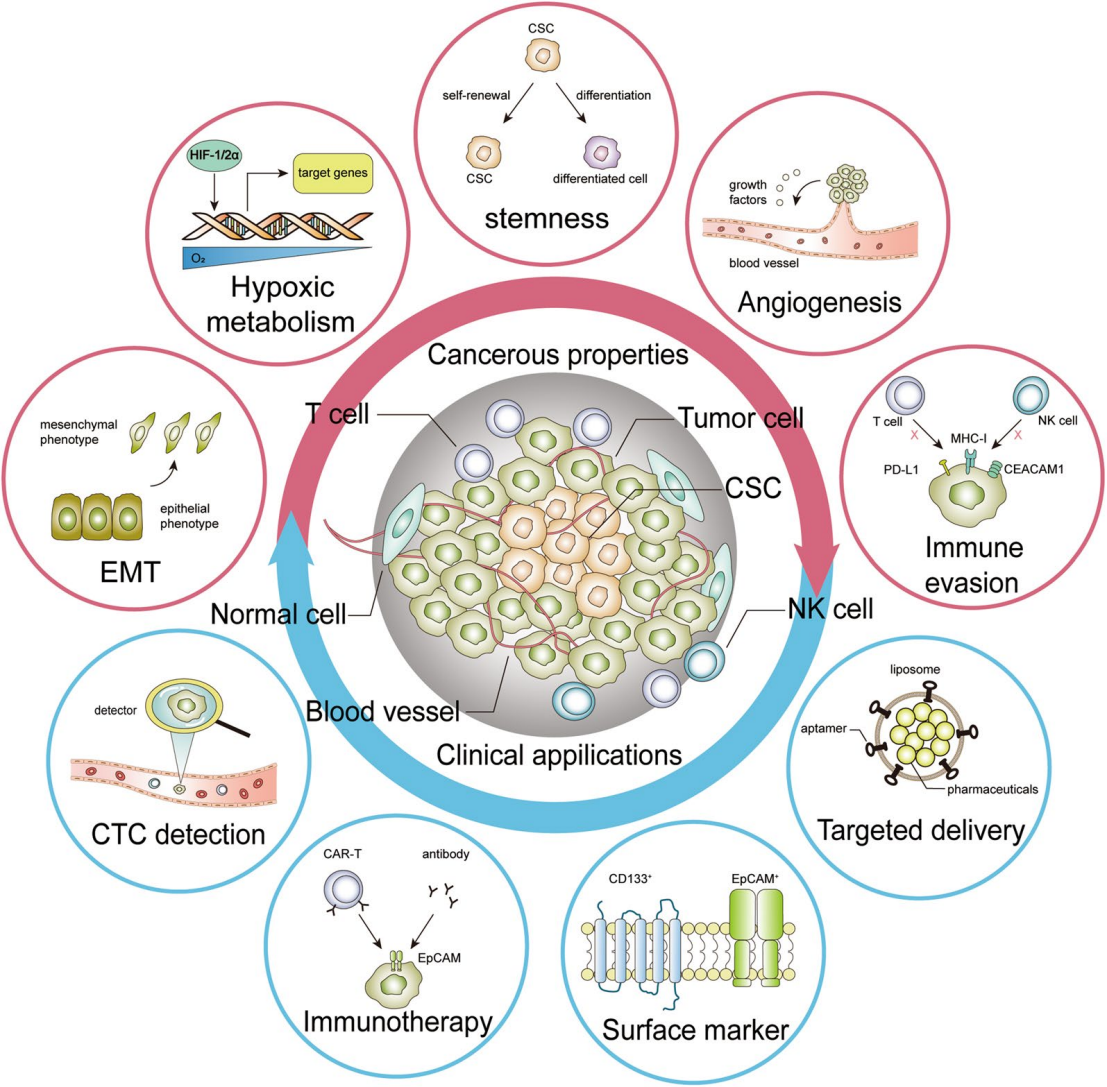
Functions and applications of EpCAM. EpCAM participates in aspects of cancer pathological processes and leads to various cancerous properties, including EMT, hypoxic metabolism, stemness, angiogenesis and immune evasion. Due to its versatile functions in cancer development, diverse clinical applications are displayed and come into use. EpCAM-targeting immunotherapy and modified microparticles have shown clinical prospects for cancer therapy. In addition, EpCAM is used for CTC detection, as well as a CSC marker for diagnosis and prognosis values

With advances in basic research, EpCAM was revealed to be a multifaceted player in cancer progression (Fig. 4). Beyond its classic role in cell adhesion, which is also context dependent, EpCAM fulfills versatile functions by meditating cell signaling from both extracellular and intracellular compartments. EpCAM orchestrates cancer progression by modulating cellular characteristics such as proliferation, stemness, mobility, chemo/radio-therapy resistance and so on. Furthermore, the immune microenvironment can also be modulated by pathological expression of EpCAM. In particular, the functions of EpCAM are highly context-dependent and dynamic. It is arbitrary to assert it as oncogenic or tumor-suppressive. Nevertheless, from the clinical perspective, EpCAM has great potential value for the diagnosis and prognosis of cancer patients. In addition to improving its sensitivity and specificity as a biomarker of CTCs, human antibodies targeting EpCAM in cancer have been applied in clinical trials. Furthermore, EpCAM expression in cancer cells provides a valuable target for precision therapy. With the increase in our knowledge of cancer characteristics, the mystery of the role of EpCAM in cancers may be further elucidated, leading to more valuable applications in cancer management.

## Data Availability

Not applicable.

## Abbreviations

ADAM17: A disintegrin and metalloproteinase 17
ADCs: Antibody–drug conjugates
ADCC: Antibody-dependent cell-mediated cytotoxicity
AML: Acute myeloid leukemia
AsiCs: Aptamer-linked small-interfering RNA chimeras
BC: Breast cancer
BiKE: Bispecific NK-cell engager
BiTE: Bispecific T-cell engager
BMP: Bone morphogenetic protein
CAR: Chimeric antigen receptor
CCND1: Cyclin D1
CD: C-terminal domain
CDC: Complement-dependent cytotoxicity
circRNAs: Circular RNAs
CRS: Cytokine release syndrome
CSCs: Cancer stem cells
CTCs: Circulating tumor cells
CTE: Congenital tufting enteropathy
DFS: Disease-free survival
DOX: Doxorubicin
DSS: Disease-specific survival
EAC: Esophageal adenocarcinoma
EGF(R): Epidermal growth factor (receptor)
EMT: Epithelial to mesenchymal transition
EpCAM: Epithelial cell adhesion molecule
EpCTF: EpCAM C-terminal fragment
EpEX: EpCAM extracellular domain
EpICD: EpCAM intracellular domain
Erα: Estrogen receptor alpha
EVs: Extracellular vesicles
FHL2: Four and a half LIM domain protein 2
HBx: Hepatitis B virus X protein
HIF-1α(2α): Hypoxia inducible factor 1α(2α)
HMECs: Human mammary epithelial cells
HNSCC: Head and neck squamous cell carcinoma
ICAM-1: Intercellular adhesion molecule 1
IGN: Indolinobenzodiazepine pseudodimer
IL-2: Interleukin-2
Lef-1: Lymphoid enhancer Factor 1
K19: Keratin 19
lncRNA: Long non-coding RNAs
MBC: Metastatic breast cancer
miRNAs: MicroRNAs
MHC-1: Major histocompatibility complex class I antigen
ND: N-terminal domain
NPC: Nasopharyngeal carcinoma
NSCLC: Non-small cell lung cancer
OS: Overall survival
OXPHOS: Oxidative phosphorylation
PAK4: P-21 activated kinase 4
PMPCB: Mitochondrial processing peptidase subunit beta
PS-2: Presenilin-2
RIP: Regulated intramembrane proteolysis
TACE: TNF-α converting enzyme
TGF-β(1): Transforming growth factor-β(1)
TM: Transmembrane domain
TriKE: Trispecific NK-cell engager
Trop2: Trophoblast cell surface antigen 2
TY: Thyroglobulin-type A1 domain
